# An Adaptable and Unsupervised TinyML Anomaly Detection System for Extreme Industrial Environments †

**DOI:** 10.3390/s23042344

**Published:** 2023-02-20

**Authors:** Mattia Antonini, Miguel Pincheira, Massimo Vecchio, Fabio Antonelli

**Affiliations:** Fondazione Bruno Kessler, Via Sommarive 18, 38123 Trento, Italy

**Keywords:** Internet of Things, TinyML, Tiny-MLOps, machine learning, anomaly detection, blockchain

## Abstract

Industrial assets often feature multiple sensing devices to keep track of their status by monitoring certain physical parameters. These readings can be analyzed with machine learning (ML) tools to identify potential failures through anomaly detection, allowing operators to take appropriate corrective actions. Typically, these analyses are conducted on servers located in data centers or the cloud. However, this approach increases system complexity and is susceptible to failure in cases where connectivity is unavailable. Furthermore, this communication restriction limits the approach’s applicability in extreme industrial environments where operating conditions affect communication and access to the system. This paper proposes and evaluates an end-to-end adaptable and configurable anomaly detection system that uses the Internet of Things (IoT), edge computing, and Tiny-MLOps methodologies in an extreme industrial environment such as submersible pumps. The system runs on an IoT sensing Kit, based on an ESP32 microcontroller and MicroPython firmware, located near the data source. The processing pipeline on the sensing device collects data, trains an anomaly detection model, and alerts an external gateway in the event of an anomaly. The anomaly detection model uses the isolation forest algorithm, which can be trained on the microcontroller in just 1.2 to 6.4 s and detect an anomaly in less than 16 milliseconds with an ensemble of 50 trees and 80 KB of RAM. Additionally, the system employs blockchain technology to provide a transparent and irrefutable repository of anomalies.

## 1. Introduction

Within the last decade, modern civilization has witnessed the birth and rise of cloud computing as one of the leading innovation enablers in the digital domain. More recently, the convergence of cloud computing with the Internet of Things (IoT) and artificial intelligence (AI) has progressively started a disruptive transformation of several industries and public sectors [1]. Today, AI tools can perform unprecedentedly well or with great accuracy in various applications, including autonomous vehicles, anomaly detection, object detection, facial recognition, and natural language processing, to name a few examples. However, these AI applications can be computationally heavy in memory, CPU, network bandwidth, etc. Furthermore, giant volumes of data will be generated by billions of connected devices [2] for these applications, to the point that it will not be possible to transfer, process, and store this data in the cloud. As an example, today, even at lower levels of autonomy, connected cars generate around 25 GB of data per hour (https://www2.deloitte.com/us/en/pages/advisory/articles/connected-vehicles-shift-an-industry.html, accessed on 24 January 2023), while, in projection, around 58 million vehicles with at least Level 3 autonomy will be sold worldwide in 2030 (https://www.statista.com/statistics/1230733/projected-sales-autonomous-vehicles-worldwide/, accessed on 24 January 2023).

These numbers suggest a dismissal of traditional cloud-centric approaches in favor of more sustainable, distributed computing paradigms [3]. One of these is edge computing [4], whose main founding principle is to process the generated data as close as possible to the source. Briefly, it complements and extends centralized cloud computing services with computing infrastructure located at the edge of the network, leveraging the computational capabilities offered by connected devices able to sense and actuate (hence, autonomously control) the surrounding environment. edge computing promises to open up new opportunities to create novel, “latency-sensitive” (i.e., more able to respond in real-time) and “context-aware” (i.e., able to adapt to the execution environment) applications. These advantages are particularly valuable for extreme industrial environments, where the operating conditions may affect communication, physical access, and safety of equipment, systems, and workers [5]. Thus, edge computing applications designed to deal with these harsh conditions can play a critical role in making the environment safer, more efficient, and more productive [6].

Notwithstanding its great potential, edge computing technologies are still far from widespread adoption since no commonly agreed solutions or approaches are available to address all the inherent challenges. Among these challenges, one of the most relevant is to “orchestrate” much more heterogeneous hardware and software toolchains than those needed in more traditional cloud platforms. Briefly, in the context of service-oriented architectures, orchestration is considered the automated configuration, coordination, and management of computer systems and software [7]. Microservice architectures have become the de facto standard design pattern to implement modern cloud applications [8]. Applications designed as loosely coupled components are easier to develop, deploy, monitor, scale, and maintain. Furthermore, infrastructure virtualization technologies (virtual machines, containers, etc.) allow the abstraction from physical resources and enable application modularization. Therefore, infrastructure virtualization technologies can be considered the primary technological enablers of microservice architectures, while the second enablers are the orchestrators themselves that help DevOps generically automate the life-cycle management. Among the solutions that have boosted this approach, Kubernetes (a.k.a. K8s) has been adopted by almost 50% of organizations worldwide in 2021 [9]. When an edge-based application relies on advanced AI tools such as machine learning/deep learning models (ML/DL), the challenges for research and innovation become even greater. Indeed, hosting AI capabilities on small and constrained devices is a technology trend that will transform all industries in the coming years by delivering more powerful and ubiquitous capabilities every day.

However, a uniform view is still lacking on what AI (ML/DL, in particular) and IoT/edge embrace. On the one hand, researchers [10] settle for the possibility of hosting ML capabilities on cheap Linux-based single-board computers (SBCs). This class of devices is indeed quite cost-attractive: for instance, a “Raspberry Pi Zero” is available off-the-shelf for less than EUR 15. The main drawbacks of SBCs [11] are related to the impossibility of upgrading the device once the CPU or the memory has been chosen. Moreover, they may require hundreds of milliwatts (mW) to run, giving them battery-powered lifetimes on the order of hours, depending on the physical size of the energy storage. For example, keeping one running for a few days requires a battery similar to a smartphone, making it challenging to build genuinely untethered experiences.

On the other hand, low-cost 32-bit MCU can host ML applications [12]. Embedded devices such as these cost less than EUR 1 but have some stringent resource constraints: they are endowed with a few KB of SRAM, have similar amounts of flash memory for persistent program and data storage, and have clock frequencies of just a few tens of MHz. Moreover, they host some non-Linux-based embedded real-time operating systems (RTOS). From an energy perspective, they only need a few mW to operate, meaning that a device running on a coin battery has an expected lifetime of the order of one year, depending on the implemented application. Indeed, these approaches can also be applied to manufacturing scenarios where the pushing of intelligence close to the production lines helps to dramatically increase the efficiency of the plant, even in legacy application contexts dated back a few decades [13].

In this paper, we design and deliver an adaptable and unsupervised anomaly detection system for extreme industrial scenarios, where the operating conditions present several challenges in terms of energy, communication, physical access, and safety [5]. We combine the edge computing paradigm with IoT best practices and AI tools to ensure reliable and safe performance. Edge computing allows local processing of data enabling real-time decision-making and monitoring. IoT devices can collect various data from the plant, and AI tools can get insights about what is happening. Leveraging these technologies allows for delivering essential monitoring functionalities even if an unexpected condition happens to a sensing device or the monitored equipment. Furthermore, an application designed to deal with these harsh conditions can play a critical role in making the environment safer, more efficient, and more productive. The proposed system is based on an IoT Kit, based on a low-cost, resource-constrained MCU that is placed a few centimeters far from the data source and, once deployed, the Kit is not physically accessible anymore. We implement a working prototype of the system for a use case where the IoT is installed under a few meters of wastewater inside an underwater pump of a wastewater management plant. The system’s objective is to monitor the run-time behavior of the rotating engine to detect anomalies that can affect the bearings, the fan, or similar pieces of equipment. Here, the environment imposes extreme operating conditions, and the embedded device is subjected to computational constraints. Nonetheless, the IoT Kit implements an ML-based unsupervised technique able to detect anomalies when the system is not behaving correctly. To this end, the device implements a complete ML pipeline (i.e., a sequence of steps to produce and infer an ML model [14]) from data sampling to feature extraction, to model training and inference, up to notifying when an anomaly occurs. We refer to this pipeline as a TinyML pipeline since it mainly runs on a constrained device. A common definition of TinyML [15] considers the execution of a neural network model by an MCU, or similar device, with a power budget lower than one mW. In our case, we consider the execution of an unsupervised ML model by a constrained device at the very far edge of the network. Finally, to make the system fully auditable and non-repudiable, the detected anomalies are transmitted by the device as signed transactions stored in a public blockchain.

Part of this study was presented in [13], where we provided the preliminary development of a TinyML anomaly detection application in the described extreme environment. The main improvements and contributions of this paper are:

- The definition of a Tiny-MLOps pipeline [16] to handle the processing workflow;
- The design of an adaptable and unsupervised anomaly detection system for extreme industrial environments.

The rest of the paper is organized as follows: Section 2 discusses the related works with the proposed system. Then, the system and the evaluation environments are detailed in Section 3 and in Section 4, respectively. The performance evaluation of the IoT Kit is presented in Section 5. Finally, Section 6 concludes the paper with final remarks.

## 2. Related Works

Hosting AI capabilities at the edge of the network represents a significant technological trend that will transform all vertical applications within the next few years. Indeed, this is strongly driving approaches to design, implement, and operate applications and devices. The initial approach has been to adopt cloud technologies such as the microservice methodology [8], in conjunction with the edge and fog computing paradigms [4], to design applications and migrate functionalities on devices, gateways, or edge servers. The work [2] depicts an anomaly detection system to detect audio anomalies in offices that exploits a micro-service-based architecture: the sensing devices perform local feature extraction and then forward the pre-processed data to the edge node that hosts the intelligence, i.e., the anomaly detection algorithm. A similar approach was proposed by Kim et al. [17] that describes an anomaly detection method based on a generative adversarial network (GAN) model to discriminate whether sensed data (i.e., images) is abnormal or not, then forwards data to another processing entity for further analysis. Here, the edge of the network contains heterogeneous computing devices (a workstation and an SBC) performing the training and the inference of the GAN model. Additionally, a low-power device performs an initial screening to identify anomalies. Instead, Shiyao et al. [18] propose and describe a federated learning framework able to collaboratively train a shared global model on edge devices. In greater detail, the edge devices execute a weakly supervised model for feature extraction by exploiting a transformer network, while a model hosted in the cloud (based on a multilayer perceptron network) performs the anomaly detection.

The evolution of application design is also pushed by the availability of new powerful computing devices able to run complex and demanding operations at the edge of the network. Özgür et al. [19] present an architecture for anomaly detection applications to monitor autonomous transfer vehicles (ATVs) during their operations. They use an edge device empowered with a GPU to infer pre-processed data to detect anomalies before sending information to another, yet more powerful, processing entity. Pham et al. [20] propose an insect monitoring system based on a trap and a vision algorithm to alert farmers when the quantity of flies exceeds a predefined threshold. This system is based on Raspberry Pi and Arduino boards embedding a YOLOv4-tiny and SSD-MobileNet deep networks. A similar approach was proposed by Saradopoulos et al. [21] that trains a DNN with a properly crafted dataset and then compares the system performance of their different edge devices (i.e., ESP32, Raspberry PI 4, and Google Coral Dev Board) in term of power consumption, accuracy, the processing time, and memory footprint. In the intelligent traffic management domain, Zhang et al. [22] adapt the YOLOv5 network to detect zebra-crossing using images captured by a camera mounted in the front of a car. An NVIDIA Jetson Nano board infers the DNN, and the authors declare that the device can deliver a detection rate of 33.1 FPS with an F1 score higher than 94%.

These applications play with the IoT, ML, and the possibilities to execute a fully fledged operating system such as Linux.

**Figure 1:**
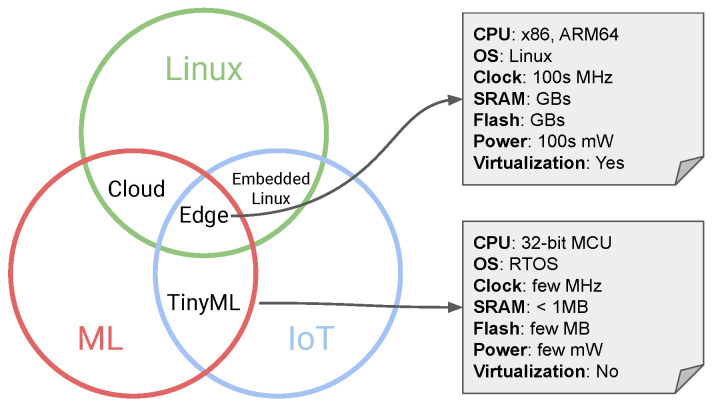
Overlapping of technological areas. Image inspired by Figure 1 in [31].

In this direction, Figure 1 shows the intersection of the different technological areas. The one we explore in this paper is the TinyML area [23], where we aim to execute an ML application on an IoT device, even if it carries only an MCU that cannot execute a traditional OS. Instead, it may execute a real-time OS [24] such as FreeRTOS, Arduino, etc. Barbiarol and Susto [25] improve the isolation forest algorithm [26] for scenarios where a decision support system (DSS) can provide a few true labels about anomalies. This can be useful for identifying the best set of classifiers (forest) with respect to performance metrics and size. Given the simplicity of the method, it can be easily applied to tiny devices. Andrade et al. [27] designed and validated a TinyML algorithm based on the TEDA [28] statistical method to detect anomalies on roads such as bumps, obstacles, potholes, and so on. Alati et al. [29] apply a multilayer perceptron (MLP) to forecast the indoor temperature of a greenhouse, with the final aim of adjusting the temperature. The model was trained in the cloud and then ported to an Arduino microcontroller connected with sensors. Xu et al. [30] developed a neural co-processor (NCP) to improve the inference of neural networks in embedded devices, while reducing power consumption. Their system can run with a power budget of ∼160 mW and a frame rate of 30 FPS.

The possibility to locally execute an ML model has also pushed research in developing algorithms and methods to even train models on tiny devices. Ren et al. [32] describe TinyML Online Learning (TinyOL), a technique to perform incremental on-device learning directly on embedded devices using streaming data. They test their approach to fine-tune a deep learning model in both supervised and unsupervised settings. On the other hand, Cai et al. [33] propose TinyTL, a method to perform transfer learning (TL) directly on devices. It exploits the freezing of network weights and updates only the biases without storing intermediate activations. The authors propose an optimized memory-efficient bias module that can learn reduced feature maps adding only a small memory overhead. Sudharsan et al. [34] propose Edge2Train, an Arduino-compatible framework to locally train, infer, and evaluate SVM models on commodity MCUs. Their approach allows for improving the running model at run-time without relying on cloud entities. Instead, in another of their papers [35], the authors present Train++, an algorithm to locally and incrementally train a TinyML model directly on an embedded device without relying on a cloud or any other external service. The target ML problem has to be reduced to a set of binary classifiers. Pau and Ambrose [12] highlight the possibilities offered by the on-device training of TinyML models by exploring the literature on neural architecture search (NAS) and the approaches to deal with concept drift. Moreover, they introduce the use of extreme learning machines (ELM) for feature extraction modules and the use of Restricted Coulomb Energy networks to learn On-Device classifiers with low-resource footprint. A comparison of the different works is presented in Table 1.

Pushing the training of models, or at least their fine-tuning phase, on embedded devices allows for delivering even more powerful Edge AI applications able to self-adapt to the target application and environment. For instance, Kayan et al. [36] propose a software framework to build a complete end-to-end processing pipeline for anomaly detection applications from the configuration of sensing devices to the training and inference of different anomaly detection models (e.g., convolutional neural networks, support vector machines, isolation forest, etc.). This step can also be easily integrated into an MLOps loop [37], a development methodology to design ML applications following a DevOps approach. In this case, given that the model is handled on a tiny embedded device, the MLOps life-cycle requires specialization for the specific class of devices; that is what we re-labeled as the Tiny-MLOps loop [16].

## 3. Proposed System

**Figure 2:**
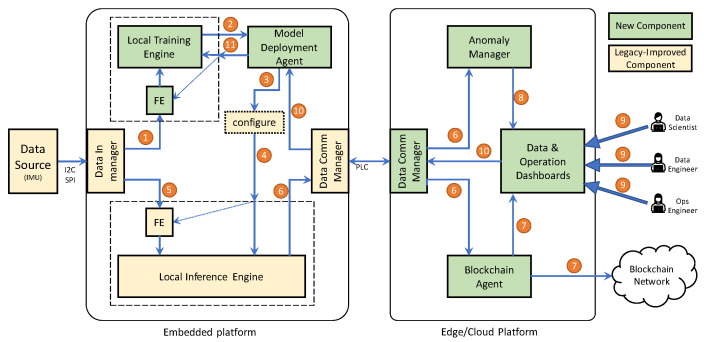
Schema of the proposed system including the Tiny-MLOps pipeline. Green blocks are the newly introduced blocks, while yellow blocks have been improved since [13].

In this section, we described our proposal for a conditioning monitor system suitable for industrial plants with extreme operational conditions. The objective is to provide a system that can adapt without prior knowledge to the target equipment and, at the same time, gain insights from the health status of the industrial system under monitoring. The proposed system is composed of two parts: an embedded platform and an edge/cloud platform, as shown in Figure 2. The system has been designed by following the Tiny-MLOps approach [16] for anomaly detection systems. This methodology allows for designing the desired pipeline and the auxiliary computing blocks useful in provisioning, maintenance, monitoring, upgrading, and improvement of the whole deployed system.

**Figure 3:**
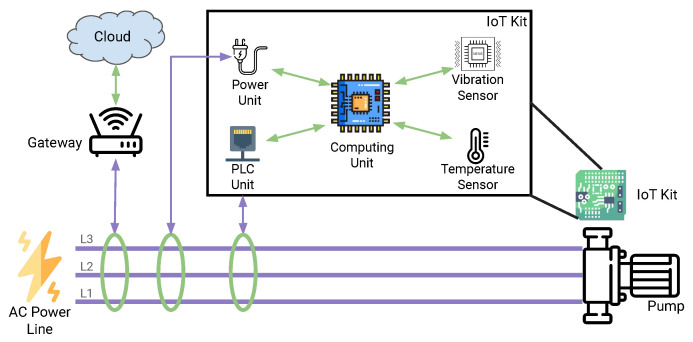
Schema of the proposed system: the rotating pump where the sensor is installed, the power line used for energy and communication, the gateway, and the cloud/external world.

The embedded platform, shown in Figure 3, is an IoT retrofitting Kit (the Kit) based on a micro-controller unit (MCU) that collects physical measures from an industrial asset through different sensors. An inertial motion unit (IMU) collects the vibration (i.e., acceleration and rotation) over the three cardinal axes, and a temperature sensor samples the temperature of the engine (i.e., the temperature inside the chassis). The Kit is deployable inside the engine chassis and is enclosed in a protective and insulating shield to avoid any short-circuit with the asset. Additionally, the device can fully exploit the pre-existing wiring to harvest energy and communicate with the outside world. To this end, the Kit hosts a small and efficient AC–DC power unit that converts and adapts the high-power AC signal to a low-voltage DC signal to feed the electronics. Furthermore, the Kit uses the same AC power wiring to convoy a high-frequency signal around 132KHz, which carries data after a proper modulation with a modem chip. The communication technology is known as power line communication (PLC) and allows a low-bit data rate (i.e., 2400 bps). This technology, compliant with the CENELEC EN 50065-1:2011 [38] specification, is also widely adopted by power distributors to remotely collect power meter readings in domestic and industrial environments without needing an on-site operator or technician. Thus, the Kit communicates with the outside world through a gateway equipped with a PLC transceiver installed on the other side of the power line. One key feature of the embedded platform is that it hosts the end-to-end processing pipeline that collects data and executes AI tasks to detect anomalies using the constrained MCU without external processing. Another key feature is that the Kit can directly interact with a blockchain system which may be used for audit operations.

On the other side of the power line channel, the edge/cloud platform is deployed on a Linux gateway based on an SBC board, i.e., a Raspberry Pi 4, installed close to the monitored asset. The gateway contains a PLC modem (i.e., an ST7540 chip) to handle the communication with the Kit via the PLC channel and the outer world using traditional network mediums (e.g., Ethernet, WiFi, 3G/4G/5G). Furthermore, the edge/cloud platform allows the deployment of new models or changes in the Kit configuration, manages the notification of anomalies, handles the telemetry from the device, and forwards anomaly information to a blockchain-based registry of anomalies. The registry relies on a public blockchain infrastructure with advanced scripting capabilities external to the proposed system. The reason for selecting a public infrastructure is two-fold: First, we create an open registry of information (in this case, anomalies), enabling the inclusion of additional actors interested in that information. This approach follows the current trend of modern blockchain-based applications using public networks o [5,39,40]. Second, by relying on external public infrastructure, we bypass the additional resources (i.e., energy, processing power, and communications) required to run the consensus algorithms and maintain the distributed ledger [41]. Although other types of infrastructure (e.g., private or consortium) and platforms (e.g., Hyperledger, Iota, Multichain) might have lower resource requirements, we favored the security provided by a public network for developing blockchain-based applications [41,42].

Starting at the embedded platform, the IoT Kit can work in two different modalities: Training Mode or Inference Mode. The Training Mode (steps 1–4) is used to understand the normal behavior of the asset under monitoring by learning a model from the collected data. Then, the Inference Mode (steps 5–6) is used when the device has already learned the normal behavior of the system and uses the model to detect possible anomalies. The working mode is selected at the boot time of the IoT Kit and depends on the status of the device and its configuration. More in detail, the steps implemented in the IoT Kit are the following:

- Boot: at boot time, the device configures the Data Source devices (e.g., IMU, temperature sensor), verifies the local configuration, and checks if it has to enter in Training Mode or Inference Mode. If it enters the Inference Mode, it loads the model from flash memory to RAM;
- Training Mode
  1. The Data In Manager buffers data (buffer size defined in the configuration) and forwards them to the Local Training Engine;
  2. The Local Training Engine computes a set of features with the feature extraction (FE) module. Then, the features are buffered up to a “critical mass”, then the model is trained. The hyper-parameters of the algorithm are defined in the device configuration. Once ready (it may take minutes), it forwards the model to the Model Deployment Agent;
  3. The Model Deployment Agent stores the model in the device flash memory and deploys the model in the Local Inference Engine;
  4. When the model is placed in the Local Inference Engine, the Inference Engine is configured by also deploying the FE module (identical to the FE module available in the Local Training Engine) required to execute the model. Once ready, the device is rebooted.
- Inference Mode
  5. The Data In Manager buffers data (buffer size defined in the configuration) and forwards samples to the Local Inference Engine. The Local Inference Engine computes a set of features with the FE module and infers the deployed model;
  6. If the Local Inference Engine detects an anomaly, it notifies the Data Communication Manager, which creates a digitally signed blockchain transaction with the anomalous data. Then, the Data Communication Manager forwards the notification (data and transaction) to an Edge/Cloud platform via a serial communication channel. In our case, the channel is a PLC channel, as discussed above.

At this point, the anomaly notification is forwarded to an edge/cloud platform in charge of the management. In greater detail:

6. The Data Communication Manager of the Edge/Cloud platform receives the notification and forwards it to the Blockchain Agent and the Anomaly Manager;
7. The Blockchain Agent forwards the signed transaction to the blockchain network, where a smart contract is responsible for storing the anomaly information in the distributed ledger. At the same time, the Agent sends a notification to the Data&Operation Dashboards;
8. The notification received by the IoT Kit is stored in the Anomaly Manager and computes the anomaly rate, which is important to understand whether the system is failing or if the anomaly is just a false positive. The output of the block is sent to the Data and Operation Dashboards for further evaluation provided by Operation Engineers (Ops Engineers);
9. The Data and Operation Dashboards are a set of dashboards developed to monitor the status of the IoT Kit and the sensorized asset. Here, an Ops Engineer can decide to re-train the model and change the configuration by sending a proper command. The update of the model may also be triggered by an automatic agent when a particular condition is met. Moreover, a Data Scientist or a Data Engineer can push a new configuration (e.g., the number of trees if the model is based on a forest of classifiers) or algorithm for the anomaly detection model;
10. If a new model or configuration is pushed into the Edge/Cloud platform from a user or by an automatic trigger, the system forwards the new configuration to the IoT Kit via a serial communication channel (i.e., a PLC channel, in our case) through the Data Communication Manager. Once received by the IoT Kit and verified that it is correct, the configuration is pushed to the Model Deployment Agent;
11. The Model Deployment Agent identifies the change in the new configuration: if it is not required, a re-train (e.g., changed detection threshold), it pushes the configuration to Local Inference Engine (steps 3 and 4), then reboots the IoT Kit. If it is necessary to re-train the model (e.g., change in the FE module, a model hyper-parameter, or similar), the Model Deployment Agent changes the working mode of the IoT Kit to Training Mode and pushes the new configuration to the Local Training Engine, which trains the new model once it has enough new data. The deployment of the model follows steps 2, 3, and 4 presented above.

The above pipeline describes the entire behavior of the system during the different working modes. Readers interested in the management of the anomaly notification can refer to steps 6, 7, and 8 during the Inference Mode.

### 3.1. Use Case

An industrial environment characterized by extreme conditions in terms of noise, humidity, temperature, or hazards, is known as an extreme environment. Wastewater management plants belong within this category, given the critical and complex operation carried out. Usually, these plants are composed of multiple pools where various treatments (e.g., sedimentation, polishing, oxidation) are carried to water before returning it to a river, lake, or to sea. Every pool is equipped with multiple underwater pumps to move water across the different stages or to mix the content. These pumps are constituted by a fan, properly designed to deal with the target water’s chemical properties (e.g., density), connected to a high-power electric engine with a shaft. Eventually, there might be a gearbox between the engine and the fan to increase torque and decrease rotational speed. The electric engines, given the target operations, may have a wide range of power from a few hundred Ws up to hundreds of KWs. Engines are connected to the power supply system, usually a few hundred meters away from the pool, through a terminal block placed in a water and chemical-proof chamber placed on top of the pump chassis.

Once the pumps (usually more than three pumps per pool) are placed in pools, they can be operated based on the real necessity of the plant. On the other hand, multiple redundant pumps guarantee the functioning of the plant even in the event of a malfunctioning pump. However, pumps remain in place until they fail or scheduled maintenance is required. This is mainly due to the high operational costs of retrieving a pump, inspecting it, performing maintenance operations, restoring the pump in the pool, and testing it. Most of the time, operations consist in replacing gaskets, bearings, or similar mechanical parts, even if they may last for a longer time. This is a common approach in traditional time-based maintenance strategies.

This kind of extreme environment and, more specifically, this use case offers the actual opportunity to deploy and validate the anomaly detection system we presented above in real settings. We will install the IoT Kit inside the terminal chamber of the pump; however, the Kit will not be physically accessible any more. In this direction, the IoT Kit has to satisfy a few requirements: from the hardware perspective, it has to fully exploit the high-power cable for both PLC communication and energy harvesting; it has to be unitedly and solidly installed inside the pump chassis to sample vibrations without introducing spurious signals or noise; it has to be electrically insulated to avoid any pump outage due to malfunctioning (e.g., short-circuit). On the software side, the main requirements of the IoT Kit are related to the capabilities to handle the communication and interaction with the gateway node using a PLC channel, which offers an available bandwidth of 2–4 kbaud in half-duplex mode (only one device can transmit at a time). Another main requirement is the ability to efficiently execute the anomaly detection pipeline, from data sampling to the classification, both in Training Mode and Inference Mode. Here, code optimization (at writing and compilation time), the static and dynamic memory management play an essential role in implementing and delivering a working system.

### 3.2. Implementation

The effective need to apply the anomaly detection system in a real-world wastewater plant has pushed an iterative design and implementation approach. Our proposed system has undergone two iterations. A prototype system was built and evaluated in a controlled environment in the first iteration. With this prototype, it was possible to simulate the normal and anomalous behaviors of the target asset [13]. In the second iteration, the system was re-engineered, and the entire processing and management pipeline was improved to be deployed within an underwater pump.

The MCU used for the Kit is an ESP32 mounted on an ESP32_WROVER_IE board. This carrier board mounts an external 4MB of SPIRAM and 4MB of flash memory, accessible by the ESP32 processor via the SPI bus. The board is connected via I2C protocol to a temperature sensor and to a TDK InvenSense ICM-20948 chip, which offers a 9-DoF (Degrees of Freedom) MEMS inertial motion unit (IMU) with a 3-axis accelerometer, gyroscope, and magnetometer. Moreover, this IMU is equipped with a processor for advanced in-chip computation. The communication between the IoT Kit and the external world is demanded by an ST7540 transceiver, enabling a half-duplex channel with an available bandwidth of up to 4.8 kbaud. The firmware for the MCU is based on the MicroPython (https://micropython.org/, accessed on 24 January 2023) framework, which offers a lean and efficient implementation of the Python 3 language for many hardware platforms, including ARM, ESP32, RP2040, etc. MicroPython runs a small interpreter that executes Python scripts stored in the MCU flash memory. This allows for an easily adaptable and modular solution by updating the stored script. First, we implemented the sensor driver libraries as MicroPython modules, making them callable from scripts. Then, we developed and coded our processing pipeline using a well-known set of Python libraries and tools, even if they offer a reduced set of functions, such as NumPy [43] and SciPy [44]. We adopted the ulab (https://github.com/v923z/micropython-ulab, accessed on 24 January 2023) implementation library to manipulate NumPy-like arrays in MicroPython. Starting from the pipeline defined in [13], we implemented the processing pipeline over the IoT Kit by performing the following tasks: the IMU unit has been configured to filter the acceleration and gyroscope signals with digital low-pass filters at 473 Hz and 361.4 Hz, respectively. Then, the IMU samples the 3-axial acceleration and gyroscope signals at 1125 Hz; we buffer them with a non-overlapping time window of 500 ms. We compute ten time-based features: the mean and the standard deviation of acceleration over the x and y directions; the mean of the gyroscope; and the energy of the acceleration over the three directions. This last feature is computed by applying the Parseval Theorem: we take the short-term Fourier transform (STFT) to the data in the time window (fs = 1125 Hz, N = 512), then we apply the squared modulus, then we sum all the frequency coefficients.

Regarding the anomaly detection algorithm, we adopted the isolation forest [26]. This algorithm creates a forest (ensemble) of classifiers assuming that the root–leaf depth, averaged among all the trees, is smaller for anomalous instances and larger for normal instances. This translates to a likelihood close to 0.0 if we infer a normal instance and close to 1.0 when the instance is anomalous. We adopted a pure Python open-source implementation (https://github.com/msimms/LibIsolationForest, accessed on 24 January 2023), and we improved it to better handle the memory allocation to suit the MicroPython execution environment. This is a critical aspect of MCUs, especially when allocating memory at run-time.

For the blockchain-based registry of anomalies, we considered Ethereum as the blockchain platform, given the maturity of the platform and the advanced scripting capabilities [39]. We used a custom blockchain library defined in [40] for implementing the blockchain operations that create digitally signed transactions in the MCU. A digitally signed transaction represents the fundamental unit of information exchange in Ethereum [39]. In our case, the transaction contains features of the sensor measurement, the parameters of the IF, a timestamp, and a boolean variable indicating that the registered measurement is an anomaly. Thus, the digital signature confirms the transaction’s integrity and authenticity, ensuring that the information was transmitted from the device without any modifications [40].

The signed transaction and the information about the anomaly are sent to the IoT gateway via the PLC communication channel. We designed and implemented a communication protocol to access and handle the PLC channel. The protocol implements: a carrier sense mechanism to understand if another device is transmitting over the channel and reducing the probability of collision, the frame structure for packets, the fragmentation of large payloads in multiple packets, and a re-transmission technique based on acknowledgment timeout. The protocol supports specific commands to handle the possibility of updating the model and/or the configuration deployed.

## 4. System Validation

**Figure 4:**
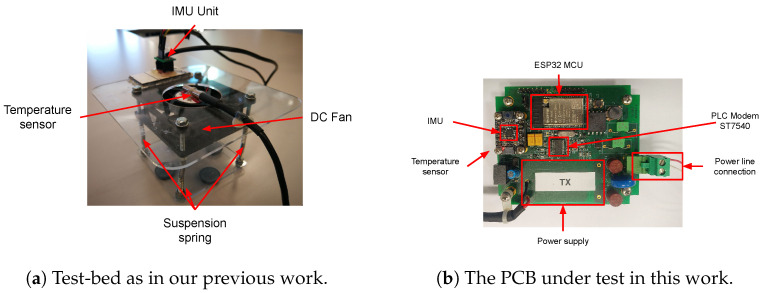
Test-bed devices built. (**a**) is the test-bed built in [13] to validate the proposed solution comprising an IMU to sample vibrations, a temperature sensor to sample the fan engine temperature, a PC 12v DC fan, and a suspended structure over springs that function asa vibrating simulator. (**b**) is the PCB board under test in this work before the deployment inside the underwater pump. The board carries the ESP32-ROVER-IE board with the MCU, the ST7540 PLC Modem, the ICM-20948 IMU mounted over a break-out board by Adafruit, the temperature sensor placed below the IMU board, the power-line connector, and the power supply unit (PSU), missing in this picture.

The validation of a system for extreme application environments may impose unexpectedly high costs that can render the system unfeasible. Thus, we need to evaluate all the components of our approach before actually deploying them in the submersible pump. A failure may happen anyway, but we can be quite sure that we reduce the points of failure. As presented in our previous work [13], the first version of the system was tested in a controlled environment where we replaced the underwater pump with a DC fan (i.e., an 80mm cooling PC fan). We firmly screwed it onto a plexiglass plate suspended over four springs, as depicted in Figure 4a.

On top of the plexiglass, we glued the IMU unit and the temperature sensor, connected to MCU, that sample the test-bed data. This test bed, when turned on, vibrates with its characteristics and can be artificially perturbed by touching it or damaging the fan to induce anomalies. This allows evaluating the actual system performance to demonstrate the approach’s applicability to real-world scenarios and improve the prototype. Using this test bed, we generated suitable datasets representative of the normal and anomalous behaviors of the system. Normal data were generated by not touching the test bed, while the artificially injected anomalies were introduced by perturbing the simulation system.

In this paper, we improved the validation approach to reach the point of having a device ready to be deployed inside a pump. The hardware components of the IoT Kit have been placed on a properly designed PCB board, as drawn in Figure 4b. It carries an ESP32_WROVER_IE processing unit (with 4MB of SPIRAM and 4MB of flash memory connected via SPI), the ICM20948 inertial unit, the temperature sensor, the AC/DC power supply unit, the PLC modem based on the ST7540 chip, and a properly designed coupling circuit to inject the PLC carrier over the power line. The PCB keeps the device compact and increases the mechanical resistance of the entire device. Indeed, the PCB can be placed inside the pump chassis with a proper insulating box/enclosure/support. Moreover, it can better sense the desired inertial signals, reducing the induced noise (e.g., the glue layer might not be flat). To evaluate the system, we installed the PCB on top of the suspended fan, then connected the IoT Kit with the gateway using a twisted-pair cable. Once the tests had been completed, the same device was properly installed inside a submersible pump to deliver the desired application. However, we cannot disseminate/show photos of the installation inside the pump due to a non-disclosure agreement.

## 5. System Evaluation

The anomaly detection system presented in this paper is based on the system originally designed in [13]. We highlight that the original system was a preliminary prototype built to prove the possibility of using MicroPython as a development environment to deliver a continuous monitoring and anomaly detection application. In the current design and implementation, as described in Section 3, we deliver multiple improvements that we evaluate in the following paragraphs. We focus on evaluating the performance of the IoT Kit, which locally trains a TinyML model to detect anomalies using data collected from target devices. Thus, we first evaluate the system performance of the IoT Kit in Inference Mode and then in Training Mode.

### 5.1. Performance in Inference Mode

The Inference Mode is the main working mode of our IoT Kit. It executes the actual anomaly detection pipeline where the model was previously trained during the Training Mode. Given that our system has to be deployed in an extreme environment where we cannot directly intervene (e.g., connect a USB cable to re-flash the firmware or push the reset button), we need to measure metrics that are important in system design [45,46], such as the memory footprint (RAM) and the model inference time (normal and anomalous instances), to ensure the Kit can remain operative for a long time; then we compare our outcome with [13]. Metrics are evaluated concerning (1) the cardinality of the ensemble of trees (size of the forest) of the isolation forest (IF) model, from 10 to 50 trees; (2) the size of the instance pool (50 or 100) sub-sampled from the training set, which as a fixed size of 100; (3) the system declares an anomaly instance if the IF normalized score is above 0.75; (4) we run 200 inferences over normal data and 200 inferences over anomalous data per configuration, then we compute the mean and the standard deviation over the single run. We expect a difference in the system metrics due to improvements in the processing pipeline. An evaluation of the detection accuracy is beyond the scope of this work.

Table 2 compares the performance of the designed systems in our previous [13] and current works. Starting from the inference time, we notice an increase in both the times required to infer a normal and an anomalous instance. This is due to the different FE modules executed by the two prototypes. Indeed, different features imply a different internal structure and exploration of the IF ensemble. Like in [13], the results highlight the behavior of the IF algorithm: the time required to explore a tree by an anomalous instance is, on average, smaller than the time required by a normal instance.

**Figure 5:**
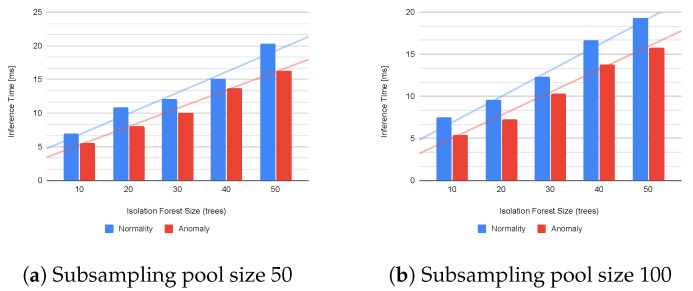
Inference delay for IF trained (training size 100) with a subsampling pool of 50 (**a**) and 100 instances (**b**). Straight lines are the trend line showing the linear dependence between the ensemble size and inference time.

Regarding the proportionality between the inference time and the ensemble size, straight lines in Figure 5 highlight the quasi-linear dependency for both the normal and anomalous instances. Moreover, the size of the subsampling pool does not impact the delays.

However, the memory footprint to run an inference with the new system is much lower. We moved from 800 KB in the case of 50 trees to just 84 KB. Like before, the memory required increases with the ensemble size but is slower than in the previous implementation. This is due to a better implementation of memory management, mainly owing to the removal of redundant variables and the additional triggering of the garbage collector. The MicroPython environment massively runs over the heap section (which has a 2 MB size) of RAM to dynamically allocate variables, functions, etc. 

When the IoT Kit enters Inference Mode, it needs to load the model from the flash memory to the RAM before inferring instances and declaring if they are anomalous or normal. For implementation reasons, the model is stored as a JSON file within the ESP32 flash memory, opened at boot time, loaded as a JSON object, then converted to an IF object. This step has a considerable computational and memory-demanding impact on the entire application’s performance that cannot be ignored. This impacts the system’s boot time since it may take seconds before the Kit becomes fully operative. Moreover, a memory estimation allows for better design of the entire application, avoiding failures due to Out-of-Memory exceptions.

Table 3 shows the measurements of the actual file size of the model over flash memory, the heap memory, and the time required to load the model (including the conversion to a JSON object and then to an IF object). Starting from the file size, it has a value of 56.9 KB (std. 3.4 KB) and 57.9 KB (std. 4.3 KB) for models with 10 trees trained with a subsampling pool size of 50 and 100 instances, respectively. File size increases by 283 KB (std. 8.3 KB and 6.1 KB) for models with 50 trees. Data show the linear dependency between file and ensemble size. The standard deviation in file size depends on the storage strategy of model parameters. We store the model as a JSON file, and consequently, all the parameters are represented as strings, including floating point numbers. Thus, the file size may increase depending on the number of digits after the unit separator. Moving to the actual loading phase, the file reading operation and string conversion to a JSON object requires ∼93 KB for the IF model with 10 trees up to ∼460 KB of heap memory, while the conversion from the JSON object to the IF object takes ∼155 KB up to ∼548 KB. This implies that the IoT Kit needs at least ∼250 KB of free heap memory to successfully load a model with 10 trees and up to ∼1 MB to load bigger ensembles with 50 trees. Once the model is loaded, it is possible to release the memory occupied by the JSON object and use it for other operations.

**Figure 6:**
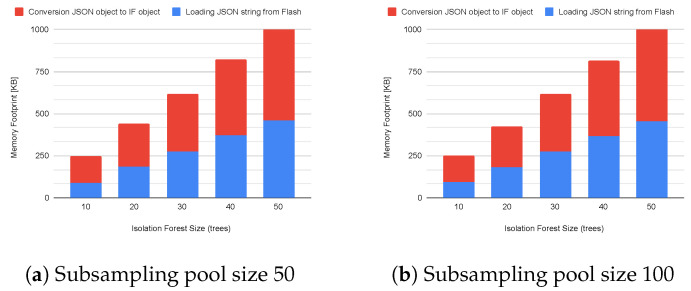
Loading memory footprint with subsampling pool of 50 (**a**) and 100 instances (**b**).

Figure 6 shows the stacked memory requirements in the case of models trained with a subsampling pool size of 50 (Figure 6a) and 100 instances (Figure 6b). Moreover, these operations take a long time to be completed due to the thousands of data operations over the flash memory and RAM. Indeed, the loading phase can take from ∼500 ms (∼340 ms for file reading and conversion to JSON object, and ∼170 ms for the object conversion) up to ∼2400 ms (∼1640 ms and ∼750 ms) for models from 10 to 50 trees, respectively. We notice that almost 2/3 of the loading time is spent reading the file and creating the JSON object due to the much lower speed of the flash memory than the RAM. However, the second phase requires more memory in the heap space.

**Figure 7:**
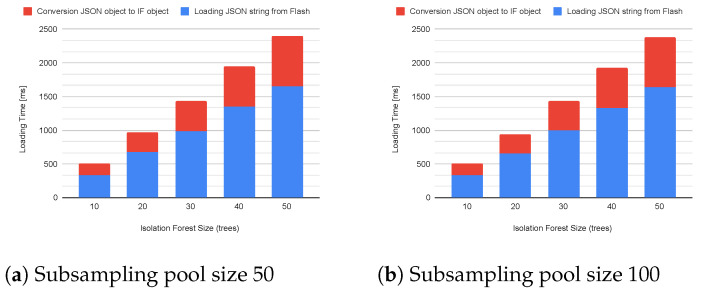
Model loading time with subsampling pool of 50 (**a**) and 100 instances (**b**).

Figure 7 shows the stack of the two loading phases.

### 5.2. Performance in Training Mode

The Training Mode is a special working mode of the IoT Kit where the device collects data to train the anomaly detection model locally. The model training is performed assuming that the target equipment is normally behaving without anomalies.

Practically, the output of the FE module is redirected from the Inference Engine to the Local Training Engine, which collects the instances until a critical mass (100 instances in our case). Then, instances are fed to the IF algorithm and, based on the subsampling pool size, randomly selects (without replacement) the actual set of instances to train the model. Finally, the algorithm builds the ensemble of trees. Finally, once the training is over, the IoT Kit stores the model parameters over the flash memory as a JSON string file.

The main operation is the actual training of the model, which requires a great quantity of memory and takes time.

Table 4 shows the memory footprint and the time required to train an IF model by varying the subsampling pool size and the cardinality of the ensemble of trees. As expected, this phase can take up to several seconds since the algorithm has to build multiple trees. The time required is ∼1.2 s for a model with 10 trees, and it goes up to ∼6.4 s for models with 50 trees. On average, the IF algorithm takes 120÷130 ms to train one classifier independently from the ensemble size. Regarding the standard deviation of the training time, it is mainly due to the depth and complexity of each tree, which is unpredictable a priori and depends on how the algorithm builds the branches. Indeed, deeper and more complex trees take more time to be built.

Regarding the memory required to train a model, we observe a strange behavior because the IF algorithm takes ∼500 KB of RAM to build a model with 10 trees, ∼1000 KB for the model with 20 trees, and ∼1500 KB for an ensemble of 30 trees. Then, the RAM required drops to ∼300 KB and ∼800 KB for models with 40 and 50 trees, respectively. Fitting a linear function with the memory requirements for ensembles of 10, 20, and 30 trees, we expect the IF algorithm training needs ∼2000 KB and ∼2500 KB to train models with 40 and 50 trees, respectively. Based on our investigation, this should be due to the garbage collector that releases memory during the model’s training. Our conjecture is also supported by the difference between the memory required for the 40 and 50 trees model training is almost the same as the memory required for 10 and 20 trees, and 20 and 30 trees model training.

### 5.3. Analysis of the Blockchain Module

Similarly to [13], we developed and implemented the Blockchain Agent following a “Smart-Twin” approach [42] and using the Ethereum platform. With this approach, a smart contract represents each industrial asset (Twin contract), while blockchain-based applications can be implemented to interact with the assets using an application contract (App Contract). Here, the device’s unique identification is mapped to a unique address on the blockchain that represents the device’s SmartTwin. For our case, the Twin Contract maintains an immutable record of measurements composed of the features, parameters, and the last time these values were updated. Furthermore, the contract maintains a count of the total measurement and the anomalies. These values come directly from the devices through a digitally signed transaction. Each transaction contains the features computed over the sensor data (10 floating numbers), the IF parameters representing the subsampling pool size and ensemble size (2 integers), the timestamp of the measurement (an integer), and a boolean variable indicating if the registered measurement is an anomaly or not.

The smart contract function first verifies if the transaction has a valid signature and belongs to the device linked to the Twin Contract. Then, the smart contract increments the internal measurements counter stores the features and parameters, and saves the current block time as the last time the contract was updated. Since storing data on the blockchain is expensive, we keep the anomaly as an event log, a less resource-consuming form of storage that aims to use external components (i.e., user interface or other computation modules). The algorithm for the update function of the Twin contract is shown in Algorithm 1.
**Algorithm 1:** Update function used in the Smart Contract.
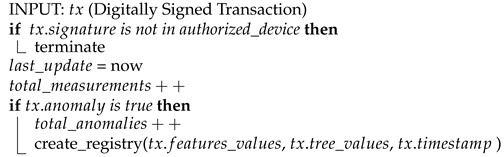


### 5.4. Transaction Size

The blockchain agent running on the embedded device generates a transaction having a size of 560 bytes. Therefore, transmitting the transaction using PLC with a maximum payload size of 200 bytes takes ∼3 s (including the non-transmitting intervals over the channel imposed by CENELEC C-band specifications [38]). This introduces an overhead of 712% compared to transmitting just the measurement information (i.e., feature values, tree values, timestamp, anomaly flag, machine UUID), which requires only 69 bytes. We have reduced the transaction overhead introduced by the previous implementation [13], where the overhead was 1043%. Even with transmission overheads, we have the guarantee that the values computed from sensor data will reach the smart contract unaltered, despite the actual hardware and software modules used to forward the information to the blockchain network.

### 5.5. Gas Consumption

Creating new information on the Ethereum blockchain (e.g., creating a smart contract, updating a smart contract) has a computational cost expressed in gas units. In the case of Ethereum, if a public network is used, these costs translate into fees for the nodes of the public infrastructure [47]. Conversely, if a private network is used, the gas can be used as a performance metric to set up the private network. Therefore, following the approach presented in [47], we measure our prototype’s gas usage and perform a monetary evaluation of the infrastructure costs for our proposed architecture.

For our evaluation, and using the model described in [47], we considered historic values during 2022, which translates into a cryptocurrency price ($P_{c}\left(m\right)$) of ETH 1 equals USD 2137 and a gas price (μ) of 10, 20, and 30 gwei for low, average, and fast processing times, respectively. Table 5 presents the computation costs of creating the Smart Contract and using the Update Function expressed in gas units plus a reference price in USD using three different values of gas prices.

Our results show that in a public blockchain network, using the historical prices of cryptocurrencies, the cost of deploying the Twin Smart contract is USD 50, and storing an anomaly costs around USD 8 in the worst case. However, using a public network does not require setting up any infrastructure and creates a truly transparent, decentralized, and fully auditable record of information. Furthermore, using the Smart-Twin approach enables the development of new decentralized applications where unknown actors can take advantage of this immutable ledger of information [39]. For instance, the OEM could deploy an application for managing guarantee procedures based on the anomalies registered in the blockchain.

### 5.6. System Scalability and Limitations

The system we presented and evaluated above is going to be installed within a submersible pump. For safety reasons, we worked on an exact replica of the prototype without the AC coupling circuit for the PLC transceiver and the AC/DC power supply unit. We used an external DC unit to power the PCB board.

Once installed in a pump and powered, the system learns the normality/anomaly model with the features computed by the pre-defined FE module, avoiding the need to have a pre-trained model. Indeed, the learned model is strictly specialized to the target pump without the “influence” of other installations. On the one hand, this opens the possibility of deploying the same hardware with the same software on different pumps without knowing the system conditions. On the other hand, the software may need some fine-tuning in terms of detection threshold, size of the ensemble, size of the training set, set of features, and so on that can be easily updated by exploiting the Tiny-MLOps methodology (Section 3). These parameters directly impact the system accuracy that cannot be estimated a priori in cases where a good amount of data is unavailable, like in the case of inaccessible assets at run-time. For this reason, we assumed that the pump is nominally working when the Kit learns the model. At the same time, the memory footprint estimation depends on the internal structure of the isolation forest ensemble built at training time. We believe that the metrics we collected should be closed, or at least in the same order of magnitude, as the actual system metrics once deployed in a pump.

## 6. Conclusions and Future Works

Nowadays, the rise of AI methods and the pervasiveness of the IoT are leading to the next generation of intelligent maintenance systems, where every piece of equipment is constantly monitored. In this way, operations can be performed only when necessary, a stringent requirement for extreme industrial environments. In this paper, we propose an adaptable condition monitoring system, originally based on [13], to monitor industrial assets, focusing on extreme environments. The proposed system is built around two main entities: an embedded IoT Kit executing a TinyML processing pipeline to detect anomalies and an edge/cloud platform to handle the telemetry and the Kit. The IoT Kit hosts the entire TinyML processing pipeline from data sampling to feature extraction to model training and inference, rendering it fully autonomous. It can train the anomaly detection model directly from data sampled by sensors without relying on any external entity; however, this requires quite a lot of computing resources, mainly to train the model, as discussed extensively in Section 5. When the Kit detects an anomaly, it sends a notification to its companion platform via constrained.The platform then performs tasks such as updating the operator’s dashboards and notifying the users. Moreover, the notification contains a digitally signed blockchain transaction that, with a blockchain infrastructure, provides auditable, transparent, and non-repudiable proof of the anomalous event.

The whole system pipeline has been designed and implemented following the Tiny-MLOps approach [16] for anomaly detection applications, making it adaptable and configurable at run time even if the Kit is not physically accessible. Our system can be further improved by integrating other TinyML algorithms, such as deep neural networks, feature extraction modules, sensors, etc. At the same time, the system may benefit from other learning paradigms, such as federated learning, where the model is collaboratively learned.

It is important to highlight that the system developed in this paper is almost ready to become a real marketable product. From the hardware perspective, we will design an enclosure to install and deploy the Kit to ensure further protection against short circuits and damage from extreme environments. From a legal point of view, we need to certify that all the components satisfy industrial regulations via an independent and external certification authority. These certifications particularly apply to communication over the PLC channel, where strict rules regulate medium access. Finally, regarding the software performance, we need to conduct a long-term run (i.e., a few months) to verify that the system remains stable over time in the target execution environment. This environment cannot be replicated in a laboratory and will help identify potential issues that impact the system’s overall performance, such as memory and energy leaks.

## Figures and Tables

**Table 1:** Summary of related works ( [13] is our previous work). Legend: **✓**: feature available, **✗**: feature not available, ∼: feature partially available. **✓**-**✗**: feature available on the first hardware platform, not available on the second hardware platform. S: Supervised Learning, U: Unsupervised Learning, F: Federated Learning.

Ref	Year	On Tiny Device	Infer on Edge	Train on Edge	Learn Type	Algorithm	Execution Platform	Validation Domain
[2]	2018	**✗**	**✓**	**✗**	U	Isolation Forest	Arduino and Linux	Office
[17]	2021	**✗**	**✓**	**✗**	S	DNN	NVIDIA Jetson Nano & PC	Industrial
[18]	2022	**✗**	**✓**	∼	S+F	DNN	PC	Toy Dataset
[19]	2022	**✗**	**✓**	**✗**	S	DNN	NVIDIA Jetson TX2	Vehicle
[20]	2021	**✗**	**✓**	**✗**	S	DNN	Arduino and Raspberry Pi	Agriculture
[21]	2022	**✓**- **✗**- **✗**	**✓**	**✗**	S	DNN	ESP32 - RaspberryPi - Coral Board	Smart Cities
[22]	2022	**✗**	**✓**	**✗**	S	DNN	NVIDIA Jetson Nano	Vehicle
[25]	2022	**✗**	∼	**✗**	U	Isolation Forest	PC	Toy Dataset
[27]	2021	**✓**	**✓**	Not Necessary	U	TEDA	Arduino Nano 33	Vehicular
[29]	2022	**✓**- **✗**	**✓**	**✗**	S	MLP	Arduino Nano 33 - Raspberry Pi 4	Agriculture
[30]	2023	**✓**	**✓**	**✗**	S	DNN	ST ARM M7 + ASIC	ImageNet
[32]	2021	**✓**	**✓**	**✓** Online	S-U	DNN- Autoencoder	Arduino Nano 33	Industrial
[33]	2020	**✗**	**✓**	**✓** Transfer Learning	S	DNN	Linux	ImageNet CIFAR10 CIFAR100
[34]	2020	**✓**	**✓**	**✓**	S	SVM	Arduino compatible	Toy Dataset
[35]	2022	**✓**	**✓**	**✓** Incremental	S	Binary Classifier	Arduino compatible	Toy Dataset
[12]	2022	**✗**	**✓**	**✓**	S	RCE-NN	Linux	Sussex Huwaei Locomotion Dataset
[13]	2022	**✓**	**✓**	**✗**	U	Isolation Forest	MicroPython ESP32	Industrial
This work		**✓**	**✓**	**✓**	U	Isolation Forest	MicroPython ESP32	Industrial

**Table 2:** Comparison between our previous work ([13]) vs. this work of the performance of the IoT Kit in Inference Mode with different sizes of the IF ensemble, different sizes of the subsampling pool, and detection threshold set to 0.75. Values A ± B show the mean (A) and the standard deviation (B).

Ensemble Size (Trees)	Subsampling Size	Inference Time [ms] Normal		Inference Time [ms] Anomaly		Memory Footprint [b]	
		Previous Work [13]	This Work	Previous Work [13]	This Work	Previous Work [13]	This Work
		Mean ± Std	Mean ± Std	Mean ± Std	Mean ± Std		
10	50	3.53 ± 1.86	6.9 ± 5.15	1.78 ± 0.5	5.5 ± 3.9	267,183	70,805
	100	3.91 ± 2.28	7.47 ± 5.28	2.00 ± 0.5	5.4 ± 3.86	360,640	71,146
20	50	5.65 ± 2.58	10.84 ± 6.44	4.48 ± 2.44	8.1 ± 5.22	789,024	73,428
	100	5.88 ± 2.68	9.61 ± 8.53	4.96 ± 2.52	7.26 ± 4.56	794,800	72,879
30	50	8.12 ± 3.16	12.1 ± 6.43	6.68 ± 2.72	10.04 ± 5.54	344,096	76,547
	100	8.333 ± 3.00	12.32 ± 6.3	6.71 ± 2.84	10.29 ± 5.89	370,192	76,180
40	50	9.97 ± 2.58	15.1 ± 6.85	8.33 ± 2.4	13.7 ± 6.9	480,640	79,433
	100	11.05 ± 3.43	16.69 ± 7.16	8.72 ± 2.8	13.8 ± 7.04	467,568	80,550
50	50	13.21 ± 3.55	20.33 ± 7.17	11.42 ± 3.16	16.27 ± 7.04	884,752	84,815
	100	14.67 ± 2.97	18.25 ± 7.66	10.9 ± 3.25	15.75 ± 7.06	810,128	82,210

**Table 3:** Memory footprint (flash memory and RAM occupation) and delay of the model loading phase during the Inference Mode.

Ensemble Size (Trees)	Subsampling Size	File Size on Flash [KB]	Loading Memory Footprint From File to JSON [KB]	Loading Memory Footprint From JSON to IF Object [KB]	Loading Time From File to JSON [ms]	Loading Time From JSON to IF Object [ms]
		Mean ± Std	Mean ± Std	Mean ± Std	Mean ± Std	Mean ± Std
10	50	56.9 ± 3.4	92.3 ± 5.4	154.4 ± 6.3	336 ± 20	168 ± 7
	100	57.9 ± 4.3	94 ± 6.8	156.8 ± 7.5	340 ± 26	171 ± 9
20	50	116.4 ± 4.1	188.3 ± 6.6	251.8 ± 8.6	677 ± 25	298 ± 10
	100	112.5 ± 5.7	181.8 ± 9.2	244 ± 9.1	656 ± 34	289 ± 12
30	50	169.5 ± 6.5	274 ± 10.4	341.9 ± 14.8	992 ± 38	436 ± 20
	100	170.1 ± 4.9	274.7 ± 7.9	342.9 ± 10.5	996 ± 30	437 ± 14
40	50	230.3 ± 8.2	372.5 ± 13.3	450.8 ± 14.2	1348 ± 48	602 ± 21
	100	227.0 ± 6.0	367.1 ± 9.8	445.6 ± 11.8	1333 ± 35	593 ± 18
50	50	283.6 ± 8.3	458.7 ± 13.5	547 ± 13.3	1653 ± 52	746 ± 21
	100	283 ± 6.1	455.9 ± 8.9	547.9 ± 13	1640 ± 35	743 ± 16

**Table 4:** IoT Kit system metrics during the Training Mode.

Ensemble Size (Trees)	Subsampling Size	Training Memory Footprint [KB]	Training Time [ms]
		Mean ± Std	Mean ± Std
10	50	536.6 ± 26.1	1225 ± 30
	100	547.2 ± 24.3	1254 ± 28
20	50	1092.4 ± 32.8	2492 ± 51
	100	1051.5 ± 42.4	2451 ± 62
30	50	1570 ± 46	3667 ± 52
	100	1576 ± 37.3	3695 ± 87
40	50	395.2 ± 85.2	5199 ± 58
	100	351.1 ± 53.5	5216 ± 48
50	50	861.6 ± 52.8	6400 ± 74
	100	824.8 ± 72	6371 ± 65

**Table 5:** Transaction gas usage with reference monetary costs using different gwei values.

Transaction (Type)	Gas (Units)	Price 10 gwei (USD)	Price 20 gwei (USD)	Price 30 gwei (USD)
Create Contract	785,229	16.8	33.6	50.3
Register Measurement	109,080	2.3	4.7	6.9
Register Anomaly	127,184	2.7	5.4	8.2
